# Fragilidade e rede social entre adultos brasileiros mais velhos: evidências do ELSI-Brasil

**DOI:** 10.11606/s1518-8787.2024058005525

**Published:** 2024-12-16

**Authors:** Karla Geovani Silva Marcelino, Luciana de Souza Braga, Fabiola Bof de Andrade, Karla Cristina Giacomin, Maria Fernanda Lima-Costa, Juliana Lustosa Torres

**Affiliations:** I Universidade Federal de Minas Gerais. Faculdade de Medicina. Programa de Pós-Graduação em Saúde Pública. Belo Horizonte, MG, Brasil; II Universidade Federal de Minas Gerais. Faculdade de Medicina. Departamento de Medicina Preventiva e Social. Belo Horizonte, MG, Brasil; III Fundação Oswaldo Cruz. Instituto René Rachou. Saúde Coletiva. Belo Horizonte, MG, Brasil; IV Prefeitura Municipal de Belo Horizonte. Secretaria Municipal de Saúde. Belo Horizonte, MG, Brasil

**Keywords:** Relações Sociais, Apoio Social, Fragilidade, Envelhecimento

## Abstract

Investigar os elementos da rede social associados à síndrome da fragilidade em adultos brasileiros mais velhos.

Foram utilizados dados da linha de base do Estudo Longitudinal da Saúde dos Idosos Brasileiros (ELSI-Brasil, 2015–2016). Definiu-se a fragilidade pelo fenótipo de Fried (perda de peso, exaustão, fraqueza, lentidão da marcha e baixo nível de atividade física). A rede social foi avaliada a partir do modelo conceitual de Berkman e Krishna (estrutura da rede social, característica dos laços da rede social, apoio social e interação social negativa). Potenciais variáveis de confusão incluíram características sociodemográficas (idade, sexo, escolaridade, cor autorreferida, renda familiar *per capita* e local de moradia) e de saúde (polifarmácia, multimorbidade, depressão, quedas, hospitalização e função cognitiva). As análises foram baseadas na regressão logística multinomial.

Entre os 8.629 participantes, 53,5% eram pré-frágeis e 9,1% frágeis. Os elementos da rede social que foram consistentemente associados à pré-fragilidade e à fragilidade são os seguintes: característica dos laços da rede social, apoio social e interação social negativa. Associação positiva foi encontrada para frequência menor que semanal de contato virtual com filhos(as) (OR = 1,15; IC95% 1,01–1,33 para pré-fragilidade e OR = 1,51; IC95% 1,13–2,02 para fragilidade) e para solidão (OR = 1,36; IC95% 1,19–1,56 para pré-fragilidade e OR=1,40; IC95% 1,12–1,75 para fragilidade). Associação negativa foi encontrada para apoio social (ajuda com empréstimos) (OR = 0,75; IC95% 0,60–0,94 para pré-fragilidade e OR = 0,54; IC95% 0,40–0,74 para fragilidade). Contudo, a percepção de críticas associou-se somente à fragilidade (OR = 1,35; IC95% 1,11–1,64).

A rede social é um elemento importante para a diminuição/prevenção da fragilidade em adultos mais velhos. Desse modo, políticas públicas, profissionais da saúde e da assistência social devem abranger a rede social de adultos mais velhos, em relação à característica dos laços da rede social, ao apoio social e à interação social negativa.

## INTRODUÇÃO

 A fragilidade é um grande desafio para a saúde pública diante do impacto negativo na funcionalidade dos indivíduos, o que pode levar à necessidade de cuidados de longa duração e (re)organização familiar ^1^^,^^2^ . É uma síndrome caracterizada pelo aumento da vulnerabilidade à baixa resposta homeostática e adaptativa diante de eventos estressores menores ^3^^,^^4^ , e. muitas vezes passa despercebida pelos profissionais de saúde. Em uma perspectiva multidimensional, a fragilidade é influenciada pela vulnerabilidade sociofamiliar, seja por ausência de apoio social, definido como limitada disponibilidade para ajudas, ou por uma rede social escassa ^3^^,^^5^ , que constitui um emaranhado escasso de relações sociais estabelecidas pelo indivíduo ^6^ . 

 Berkman e Krishna ^6^ , a partir de teóricos como Durkheim e Bowlby, propuseram um modelo conceitual sobre o impacto da rede social na saúde, a partir de uma estrutura macrossocial. Esse modelo estabelece que as redes sociais estão inseridas em um contexto socioeconômico, político e cultural mais amplo que determina a própria estrutura (tamanho, densidade, distância, entre outros) e a característica dos laços da rede social (frequência de contatos presencial ou virtual, reciprocidade, intimidade, entre outros), oportunizando mecanismos psicossociais que podem impactar a saúde. Entre os mecanismos, destaca-se o apoio social (impacto positivo) e as interações sociais negativas (impacto negativo). 

 A nível internacional, dados longitudinais de mexicanos com 65 anos ou mais, que vivem nos Estados Unidos, mostraram que o aumento do apoio social emocional, definido como demonstração de cuidado e afeto pelo outro, esteve associado a uma menor progressão da fragilidade entre aqueles moderadamente frágeis ^7^ . 

 Estudos longitudinais e transversal reportaram uma associação positiva entre ausência de companheiro ^8^^,^^9^ e solidão ^3^ , e pré-fragilidade e fragilidade. Quanto à característica dos laços da rede social, a literatura aponta que a frequência mensal ou inferior de contatos com amigos ^10^ , bem como uma rede social com poucas trocas associam-se a maior prevalência de fragilidade ^5^ . Um estudo longitudinal brasileiro, realizado com pessoas com 65 anos ou mais, não encontrou associação entre apoio social e fragilidade ^11^ , mas essa associação foi verificada em um estudo transversal ^12^ . Entretanto, os estudos brasileiros não foram baseados em amostra representativa nacional e não avaliaram a rede social. 

Vários estudos utilizaram a terminologia “rede social” como sinônimo de apoio social, não distinguindo os possíveis mecanismos de associação com a saúde. Dessa forma, este estudo partiu do pressuposto de que a rede social (estrutura da rede social, característica dos laços da rede social e apoio social) pode atuar como fator de proteção ou fator de risco (interação social negativa) para a fragilidade. Sendo assim, o objetivo deste trabalho foi investigar os elementos da rede social associados à fragilidade em adultos brasileiros mais velhos.

## MÉTODOS

### Desenho do Estudo

 Trata-se de um estudo transversal, com dados da linha de base do Estudo Longitudinal da Saúde dos Idosos Brasileiros (ELSI-Brasil), realizada em 2015–2016. O ELSI-Brasil é um estudo de coorte, conduzido em amostra representativa da população brasileira ≥50 anos. Todos os residentes com 50 anos ou mais, dos domicílios selecionados, foram elegíveis a participar, totalizando 9.412 participantes. Mais detalhes sobre o processo de seleção da amostra podem ser consultados em publicação anterior ^13^ . O ELSI-Brasil foi aprovado pelo comité de ética da Fundação Oswaldo Cruz-Minas Gerais (número: 34649814.3.0000.5091). Todos os participantes assinaram termos de consentimento livre e esclarecido. 

### Variável Dependente

 A fragilidade foi definida pelo fenótipo de Fried e colaboradores ^14^ , de acordo com o número de critérios positivos: três ou mais como “frágil”, um ou dois como “pré-frágil” e nenhum como “não frágil”. Os critérios foram: (1) Perda de peso: autorrelato de perda de peso de 4,5 kg ou mais nos últimos três meses, sem qualquer intenção/dieta; (2) Exaustão: frequências superiores a 3-4 dias para qualquer uma das seguintes perguntas do questionário de depressão do *Center for Epidemiological Studies* (CES-D) ^15^ . “Na última semana, com que frequência o(a) Sr(a) sentiu que não conseguiria levar adiante suas coisas (iniciava alguma coisa, mas não conseguia terminar)?”; “Na última semana, com que frequência a realização de suas atividades rotineiras exigiram do(a) Sr(a) um grande esforço para serem realizadas?”; (3) Fraqueza: força da preensão palmar no quintil inferior, após ajuste por sexo e quartis do índice de massa corporal (IMC), e aqueles na condição de acamado ou inabilidade de realizar o teste ^16^ . A força foi avaliada em três tentativas, utilizando-se um dinamômetro manual no membro superior dominante e considerando-se o melhor desempenho; (4) Lentidão da marcha: quintil de maior tempo gasto para caminhar 3 m de forma usual, estratificado por sexo e altura e aqueles na condição de acamado ou inabilidade de realizar teste ^16^ ; e (5) Baixa atividade física: quintil inferior de gasto energético mensurado em quilocalorias (kcals) semanal, estratificado por sexo ^16^ . As kcals gastas na última semana em atividades desenvolvidas no trabalho, ir de um lugar a outro, lazer, esporte, exercício ou afazeres domésticos foram contabilizadas considerando a intensidade (leves, moderadas e vigorosas) e tempo (minutos/horas), com base no *Short Form of the International Physical Activity Questionaire* (IPAQ) ^17^ . 

### Variáveis Independentes

 As variáveis independentes foram aquelas relativas aos elementos da rede social, a partir do modelo conceitual de Berkman e Krishna ^6^ sobre o impacto da rede social na saúde. Os elementos selecionados para este estudo foram: estrutura da rede social, característica dos laços da rede social, apoio social e interação social negativa. 

### Estrutura da Rede Social

 Segundo o modelo conceitual de Berkman e Krishna ^6^ , a estrutura da rede social pode ser avaliada por tamanho, densidade, distância, entre outros. Este estudo focou no tamanho da rede social e no tipo de arranjo domiciliar. 

O tamanho da rede social foi avaliado somando-se o número total de pessoas que fazem parte da rede social do participante, considerando filhos, netos ou bisnetos, irmãos e/ou irmãs vivos, a partir de três perguntas sobre quantitativo de (1) filhos vivos, (2) netos ou bisnetos vivos e (3) irmãos(ãs) vivos(as).

Para o tamanho da rede social, não foram incluídos amigos(as) e/ou vizinhos(as), pois não consta o quantitativo de pessoas para os vínculos em questão na linha de base do ELSI-Brasil. O tamanho total da rede social foi utilizado como variável contínua e, devido à sua distribuição, foi truncado em 50 pessoas. Já o arranjo domiciliar foi avaliado por autorrelato, considerando-se morar sozinho(a), morar com companheiro(a) ou outros arranjos.

### Característica dos Laços da Rede Social

 Segundo o modelo conceitual de Berkman e Krishna ^6^ , a característica dos laços da rede social pode ser avaliada a partir da frequência dos contatos (presencial ou virtual), reciprocidade, multiplicidade, duração e intimidade. Este estudo focou na frequência de contatos. 

A frequência de contato com a rede social foi definida separadamente para aqueles do tipo presencial e virtual, considerando-se somente os indivíduos que não moravam com o participante. “Contato presencial” foi definido como encontros presenciais, e “contato virtual” foi definido como conversas por telefone, Skype, WhatsApp, Facebook, com filhos(as), parentes ou amigos(as). A frequência de contato foi avaliada pelo autorrelato, conforme três categorias de resposta: pelo menos uma vez/semana, menos que uma vez/semana ou não tinha o vínculo referente à categoria analisada (filhos(as), parentes ou amigos(as)).

### Apoio Social

O apoio social instrumental foi avaliado por autorrelato da disponibilidade de ajuda: com a casa (sim ou não); com compras, pagar contas ou ir ao banco (sim ou não), caso haja um motivo de doença; e com empréstimos, incluindo dinheiro ou objetos (sim ou não). O apoio social emocional foi avaliado pelo autorrelato da disponibilidade de pessoa para confidências (sim ou não).

### Interação Social Negativa

Avaliada a partir da percepção do indivíduo referente: à solidão, à críticas e ao excesso de cuidados. Solidão, avaliada a partir da percepção em se sentir sozinho ou solitário, por pergunta única: “Com que frequência o(a) Sr(a) se sente sozinho (solitário)?” (nunca, algumas vezes ou sempre). Percepção de críticas avaliada, pela pergunta: “O(a) Sr.(a) acha que as pessoas lhe fazem muitas cobranças ou exigências ou críticas?” (nunca, algumas vezes ou sempre). Percepção de excesso de cuidados, por meio da pergunta: “O(a) Sr(a) fica incomodado(a) porque acha que as pessoas tentam ajudá-lo(a) mais do que o(a) Sr(a) acha que precisa?” (nunca, algumas vezes ou sempre). Para as três variáveis, as categorias de resposta “algumas vezes” e “sempre” foram agrupadas.

### Potenciais Variáveis de Confusão

 Foram consideradas potenciais variáveis de confusão as características sociodemográficas e relacionadas à saúde. As variáveis sociodemográficas foram: idade (50–59; 60–69; 70–79; ≥ 80 anos); sexo (feminino ou masculino); escolaridade, em anos completos (nunca estudou, 1–4 anos, 5–8 anos, ≥ 9 anos); cor autorreferida (branca, preta, parda ou outra); renda familiar *per capita* , em tercis [inferior (até R$ 558,70), médio (de R$ 558,71 até R$ 1.000,00) e superior (≥ R$ 1.000,01)]; e local de moradia (urbano ou rural). As características relacionadas à saúde foram: autopercepção de saúde (muito boa/boa; regular ou ruim/muito ruim); polifarmácia, considerando-se o uso regular de cinco ou mais medicamentos receitados por um médico ^18^ e utilizados nas duas últimas semanas (sim ou não); multimorbidade ^19^ (sim ou não); diagnóstico médico de depressão (sim ou não); autorrelato de quedas nos últimos 12 meses (sim ou não); hospitalização nos últimos 12 meses, considerando internações hospitalares por pelo menos 24 horas (sim ou não) e função cognitiva. 

 Considerou-se multimorbidade a presença de duas ou mais condições crônicas, incluindo doenças cardiovasculares (hipertensão arterial, acidente vascular cerebral, infarto agudo do miocárdio, angina e insuficiência cardíaca), doença renal crônica, doença neurológica crônica (doença de Alzheimer e doença de Parkinson), doença respiratória crônica (enfisema, doença pulmonar obstrutiva crônica e bronquite), diabetes, artrite, asma, câncer e obesidade. Todas as condições crônicas foram obtidas por autorrelato de histórico de diagnóstico médico, exceto a obesidade, que foi caracterizada com base na medida objetiva de peso e altura a partir do cálculo do IMC (≥ 30 kg/m2 para aqueles com menos de 60 anos, e ≥ 27 kg/m2 para aqueles com 60 anos ou mais) ^19^ . A função cognitiva foi avaliada a partir da linguagem e da função executiva, pelo teste de fluência verbal semântica de um minuto, considerando-se o número total de animais mencionados ^20^ . 

### Análise Estatística

As distribuições de frequência foram calculadas para as variáveis categóricas, observando-se as diferenças pelo teste qui-quadrado de Pearson, com correção de Rao-Scott. Para as variáveis contínuas, foram calculados a média e o intervalo de confiança de 95% (IC95%). As diferenças entre as categorias de fragilidade foram avaliadas pelo teste de Wald ajustado.

 Para as análises por grupos e ajustadas, utilizaram-se modelos de regressão logística multinomial para estimar *odds ratio* (OR) e seus respectivos IC95% da associação entre rede social e fragilidade. As análises foram realizadas separadamente por blocos de variáveis da rede social e, posteriormente, ajustadas por todas as potenciais variáveis de confusão, da seguinte maneira: (1) estrutura da rede social; (2) característica dos laços da rede social (contato presencial); (3) característica dos laços da rede social (contato virtual); (4) apoio social; e (5) interação social negativa. O teste de multicolinearidade foi utilizado para testar a correlação entre as variáveis incluídas nos modelos multivariados. As variáveis relativas à característica dos laços da rede social (frequência de contato) associadas à fragilidade foram plotadas em uma figura. Todas as análises foram realizadas no software Stata/SE® ( *StataCorp.* , *CollegeStation* , Estados Unidos), versão 14.2, considerando o delineamento da amostra e os pesos dos participantes. 

## RESULTADOS

 Dos 9.412 participantes da linha de base do ELSI-Brasil, 8.629 (91,7%) tinham informações completas para a classificação da fragilidade e foram incluídos. A média da idade foi de 62,2 anos (IC95% 61,4–63,1). Entre os participantes, 53,5% (IC95% 51,8–55,1) eram pré-frágeis e, 9,1% (IC95% 8,0–10,2), frágeis. As características dos participantes segundo a fragilidade estão descritas na Tabela 1 . Entre os frágeis, 50,8% tinham idade inferior a 70 anos, 44,8% até quatro anos de escolaridade e 34,0% autopercepção de saúde ruim/muito ruim. Todas as características apresentaram diferença significativa entre as categorias de fragilidade, exceto local de moradia. 

**Tabela 1.** Distribuição das características sociodemográficas e relacionadas à saúde, total e segundo classificação da fragilidade (ELSI-Brasil, 2015-2016).

**VARIÁVEIS**	**CLASSIFICAÇÃO DA FRAGILIDADE**				
	**TOTAL**	**Não Frágil**	**Pré-Frágil**	**Frágil**	**Valor de** p
**SOCIODEMOGRÁFICAS**					
**Idade (%)**					< 0,001
50–59 anos	48,3	55,7	47,3	23,7	
60–69 anos	29,9	31,0	29,6	27,1	
70–79 anos	15,4	11,3	16,3	26,6	
≥ 80 anos	6,4	2,0	6,8	22,6	
**Sexo feminino (%)**	53,7	50,8	55,3	56,2	0,007
**Escolaridade (%)**					< 0,001
Nunca estudou	12,4	7,5	13,5	26,0	
1–4 anos	38,4	33,2	41,0	44,8	
5–8 anos	21,9	23,7	21,1	19,0	
≥ 9 anos	27,3	35,6	24,4	10,2	
**Cor autorreferida (%)**					0,045
Branca	42,8	45,1	41,6	40,4	
Preta	9,6	8,3	10,2	11,6	
Parda	44,7	44,0	45,3	43,8	
Outra	2,9	2,6	2,9	4,2	
**Renda familiar per capita (%)**					< 0,001
Tercil inferior	31,6	26,8	33,7	39,0	
Tercil médio	32,9	32,0	33,0	36,4	
Tercil superior	35,5	41,2	33,3	24,6	
**Local de moradia urbano (%)**	84,8	85,9	84,2	83,6	0,399
**RELACIONADAS À SAÚDE**					
**Autopercepção de saúde, (%)**					< 0,001
Muito boa/boa	43,8	56,3	38,9	20,6	
Regular	44,8	39,4	48,5	45,4	
Ruim/muito ruim	11,4	4,3	12,6	34,0	
**Polifarmácia** ^a^ **(%)**	13,1	7,5	14,7	26,9	< 0,001
**Multimorbidade** ^b^ **(%)**	55,9	48,6	59,3	67,0	< 0,001
**Depressão (%)**	18,6	12,4	20,6	32,3	< 0,001
**Queda nos últimos 12 meses (%)**	21,8	15,7	23,6	37,0	< 0,001
**Função cognitiva média (IC95%)**	11,8 (11,5–12,1)	13,1 (12,8–13,5)	11,5 (11,2–11,8)	8,2 (7,7–8,8)	< 0,001
**Hospitalização nos últimos 12 meses (%)**	9,8	5,6	10,7	21,9	< 0,001
**N total**	8.629	3.117	4.655	857	

IC95%: intervalo de confiança de 95%.

a Uso de cinco ou mais medicamentos de forma regular

b Duas ou mais condições crônicas, incluindo: doenças cardiovasculares (hipertensão arterial, acidente vascular cerebral, infarto agudo do miocárdio, angina e insuficiência cardíaca), doença renal crônica, doença neurológica crônica (doença de Alzheimer e doença de Parkinson), doença respiratória crônica (enfisema, doença pulmonar obstrutiva crônica e bronquite), diabetes, artrite, asma, câncer e obesidade

** Valor de *p*: **teste qui-quadrado de Pearson com correção de Rao-Scott para variáveis categóricas e teste de Wald ajustado para variáveis contínuas.

 A distribuição dos elementos da rede social segundo a fragilidade está descrita na Tabela 2 . Os participantes apresentaram, em média, uma rede social de 13 pessoas (IC95% 12,4–13,8), e cerca de 65% moravam com companheiro(a). Entre os frágeis, destacam-se o maior tamanho médio da rede social (16,1; IC95% 14,8–17,4) e a maiores frequências de contato, menos que uma vez/semana, presencial (67,9%) e virtual (63,0%) com parentes. Também é possível observar que, neste grupo, há menores prevalências de apoio social instrumental (disponibilidade de ajuda com empréstimo) e emocional (disponibilidade de pessoa para confidências), maiores prevalências de solidão (58,8%) e percepção de excesso de cuidados (39%). 

**Tabela 2.** Distribuição da estrutura e característica dos laços da rede social, apoio social e interação social negativa, total e segundo classificação da fragilidade (ELSI-Brasil, 2015–2016).

**VARIÁVEIS REDE SOCIAL**	**CLASSIFICAÇÃO DA FRAGILIDADE**				
	**Total**	**Não Frágil**	**Pré-Frágil**	**Frágil**	**Valor de** p
**ESTRUTURA DA REDE SOCIAL**					
**Tamanho da rede social, média (IC95%)**	13,0 (12,4–13,5)	11,8 (11,2–12,4)	13,2 (12,7–13,7)	16,1 (14,8–17,4)	< 0,001
**Arranjo domiciliar (%)**					< 0,001
Morar sozinho(a)	8,6	7,4	9,2	9,6	
Morar com companheiro(a)	64,7	69,5	62,9	56,1	
Outros arranjos	26,7	23,1	27,9	34,3	
**CARACTERÍSTICA DOS LAÇOS DA REDE SOCIAL**					
**Frequência de contato presencial com filhos(as)**					< 0,001
Pelo menos uma vez/semana	48,3	47,7	48,5	49,7	
Menos que uma vez/semana	28,5	26,2	29,4	32,3	
Não tem filhos(as)	23,2	26,1	22,1	18,0	
**Frequência de contato presencial com parentes**					< 0,001
Pelo menos uma vez/semana	34,6	38,9	33,1	25,3	
Menos que uma vez/semana	62,0	59,1	63,2	67,9	
Não tem parentes	3,4	2,0	3,7	6,8	
**Frequência de contato presencial com amigos(as)**					< 0,001
Pelo menos uma vez/semana	69,7	72,9	68,8	62,0	
Menos que uma vez/semana	20,1	19,7	19,9	23,1	
Não tem amigos(as)	10,2	7,4	11,3	14,9	
**Frequência de contato virtual com filhos(as)**					< 0,001
Pelo menos uma vez/semana	56,7	58,3	57,0	48,0	
Menos que uma vez/semana	20,0	15,6	20,8	33,9	
Não tem filhos(as)	23,3	26,1	22,2	18,1	
**Frequência de contato virtual com parentes**					< 0,001
Pelo menos uma vez/semana	45,8	53,2	43,3	30,2	
Menos que uma vez/semana	50,8	44,8	52,9	63,0	
Não tem parentes	3,4	2,0	3,8	6,8	
**Frequência de contato virtual com amigos(as)**					< 0,001
Pelo menos uma vez/semana	43,4	49,9	41,8	25,6	
Menos que uma vez/semana	46,3	42,7	46,8	59,1	
Não tem amigos(as)	10,3	7,4	11,4	15,3	
**APOIO SOCIAL**					
APOIO SOCIAL INSTRUMENTAL					
Disponibilidade de ajuda com a casa (%)	97,4	98,3	96,7	97,0	< 0,001
Disponibilidade de ajuda com compras, pagar contas ou ir ao banco (%)	98,4	99,0	97,9	98,3	0,004
Disponibilidade de ajuda com empréstimos (%)	90,2	92,8	89,0	84,8	< 0,001
APOIO SOCIAL EMOCIONAL					
Disponibilidade de pessoa para confidências (%)	92,3	93,4	91,5	91,2	0,046
**INTERAÇÃO SOCIAL NEGATIVA**					
Solidão algumas vezes/sempre (%)	47,2	39,5	51,2	58,8	< 0,001
Percepção de críticas algumas vezes/sempre (%)	47,0	45,4	48,1	47,6	0,111
Percepção de excesso de cuidados algumas vezes/sempre (%)	32,0	29,7	32,7	39,0	0,001
**N total**	8.629	3.117	4.655	857	

IC95%: intervalo de confiança de 95%.

Nota: Valor de p: teste qui-quadrado de Pearson com correção de Rao-Scott para variáveis categóricas e teste de Wald ajustado para variáveis contínuas.

 Os resultados da regressão logística multinomial estão descritos na Tabela 3 . Como a multicolinearidade não foi evidenciada [Fator de Inflação de Variância (VIF) < 2], todas as variáveis de confusão foram mantidas nos modelos ajustados. Considerando os modelos ajustados, a chance de pré-fragilidade foi maior entre aqueles que relataram frequência de contato virtual com filhos(as) (OR = 1,15; IC95% 1,01–1,33) e parentes (OR = 1,18; IC95% 1,05–1,34) menos que uma vez/semana, não tinham parentes ou amigos e relataram sentir solidão algumas vezes/sempre (OR = 1,36; IC95% 1,19–1,56). Também observamos que a chance de pré-fragilidade foi menor entre aqueles que relataram disponibilidade de ajuda com a casa (OR = 0,66; IC95% 0,46–0,94) e com empréstimos (OR = 0,75; IC95% 0,60–0,94). 

**Tabela 3.** Resultados dos modelos por grupo e ajustados da associação entre estrutura e característica dos laços da rede social, apoio social e interação social negativa, total e segundo classificação da fragilidade (ELSI-Brasil, 2015–2016).

**VARIÁVEIS**	**Modelos por grupo**		**Modelos ajustados**	
	**Pré-frágil OR (IC95%)**	**Frágil OR (IC95%)**	**Pré-frágil OR (IC95%)**	**Frágil OR (IC95%)**
**ESTRUTURA DA REDE SOCIAL** ^a^				
**Tamanho da rede social**	1,02 (1,00–1,02)	1,05* (1,04–1,06)	1,00 (0,99–1,00)	0,99 (0,98–1,00)
**Arranjo domiciliar (versus morar sozinho(a))**				
Morar com companheiro(a)	0,75* (0,64–0,86)	0,65* (0,48–0,86)	0,87 (0,75–1,01)	0,90 (0,65–1,25)
Outros arranjos	1,00 (0,83–1,22)	1,09 (0,76–1,58)	1,06 (0,85–1,32)	1,03 (0,67–1,58)
**CARACTERÍSTICA DOS LAÇOS DA REDE SOCIAL** ^b,c^				
**Frequência de contato presencial com filhos(as) (versus pelo menos uma vez/semana)**				
Menos que uma vez/semana	1,07 (0,94–1,22)	1,09 (0,85–1,41)	1,04 (0,90–1,21)	0,93 (0,72–1,19)
Não tem filhos(as)	0,84* (0,71–0,99)	0,67* (0,49–0,91)	1,01 (0,85–1,20)	1,15 (0,81–1,63)
**Frequência de contato presencial com parentes (versus pelo menos uma vez/semana)**				
Menos que uma vez/semana	1,19* (1,07–1,33)	1,58* (1,28–1,95)	1,10 (0,97–1,25)	1,16 (0,93–1,43)
Não tem parentes	2,14* (1,55–2,95)	4,99* (2,92–8,51)	1,73* (1,21–2,48)	2,73* (1,46–5,10)
**Frequência de contato presencial com amigos(as) (versus pelo menos uma vez/semana)**				
Menos que uma vez/semana	1,06 (0,91–1,22)	1,36* (1,03–1,80)	1,00 (0,84–1,18)	1,05 (0,78–1,42)
Não tem amigos(as)	1,55* (1,30–1,86)	2,20* (1,62–3,00)	1,24* (1,04–1,48)	1,35 (0,92–1.98)
**Frequência de contato virtual com filhos(as) (versus pelo menos uma vez/semana)**				
Menos que uma vez/semana	1,20* (1,04-1.39)	1,87* (1,48-2,36)	1,15* (1,01-1,33)	1,51* (1,13–2,02)
Não tem filhos(as)	0,89 (0,76–1,04)	0,89 (0,67–1,18)	1,04 (0,89–1,22)	1,40* (1,02–1,93)
**Frequência de contato virtual com parentes (versus pelo menos uma vez/semana)**				
Menos que uma vez/semana	1,32* (1,19–1,46)	1,71* (1,36–2,16)	1,18* (1,05–1,34)	1,25 (0,95–1,63)
Não tem parentes	2,11* (1,49–2,98)	4,11* (2,43–6,96)	1,77* (1,22–2,56)	2,49* (1,41–4,41)
**Frequência de contato virtual com amigos(as) (versus pelo menos 1 vez/semana)**				
Menos que uma vez/semana	1,15* (1,00–1,32)	1,98* (1,58–2,50)	0,98 (0,84–1,15)	1,12 (0,84–1,49)
Não tem amigos(as)	1,62* (1,35–1,96)	2,95* (2,12–4,10)	1,23* (1,01–1,50)	1,36 (0,90–2.05)
**APOIO SOCIAL** ^d^				
APOIO SOCIAL INSTRUMENTAL				
Disponibilidade de ajuda com casa ( *versus* não)	0,64* (0,45–0,91)	0,68 (0,38–1,22)	0,66* (0,46–0,94)	0,67 (0,29–1,53)
Disponibilidade de ajuda com compras, pagar contas ou ir ao banco ( *versus* não)	0,63 (0,37–1,05)	0,92 (0,42—2,03)	0,62 (0,35–1,10)	1,13 (0,46–2,74)
Disponibilidade de ajuda com empréstimos ( *versus* não)	0,68* (0,56–0,83)	0,44* (0,34–0,57)	0,75* (0,60–0,94)	0,54* (0,40–0,74)
APOIO SOCIAL EMOCIONAL				
Disponibilidade de pessoa para confidências ( *versus* não)	0,89 (0,71–1,11)	0,88 (0,57–1,36)	0,95 (0,73–1,20)	0,93 (0,60–1,46)
**INTERAÇÃO SOCIAL NEGATIVA** ^e^				
Solidão algumas vezes/sempre ( *versus* nunca)	1,60* (1,41–1,82)	2,12* (1,74–2,59)	1,36* (1,19–1,56)	1,40* (1,12–1,75)
Percepção de críticas algumas vezes/sempre ( *versus* nunca)	0,98 (0,88–1,09)	0,89 (0,74–1,06)	1,09 (0,98–1,23)	1,35* (1,11–1,64)
Percepção de excesso de cuidados algumas vezes/sempre ( *versus* nunca)	1,07 (0,95–1,20)	1,39* (1,11–1,74)	0,94 (0,83–1,06)	0,93 (0,70–1,23)

OR: *odds ratio* .

IC 95%: Intervalo de confiança de 95%.

Nota: Modelos baseados em regressão logística multinomial, com categoria de referência não frágil. Modelos ajustados por idade, sexo, escolaridade, cor autorreferida, renda familiar *per capita* , local de moradia, autopercepção da saúde, polifarmácia, multimorbidade, depressão, queda, função cognitiva, hospitalização.

a  n final = 7.802.

b n final = 8.002.

c n final = 7.902.

d n final = 7.086.

e n final = 7.220.

* p < 0,05.

Já a chance de fragilidade foi maior entre aqueles que relataram frequência de contato virtual com filhos(as) (OR = 1,51; IC95% 1,13–2,02) menos que uma vez/semana, não tinham filhos ou parentes, relataram solidão (OR = 1,40; IC 95% 1,12–1,75) e percepção de críticas (OR = 1,35; IC95% 1,11–1,64) algumas vezes/sempre. Por outro lado, menores chances de fragilidade foram encontradas para disponibilidade de ajuda com empréstimos (OR = 0,54; IC95% 0,40–0,74).

**Figura 1.**
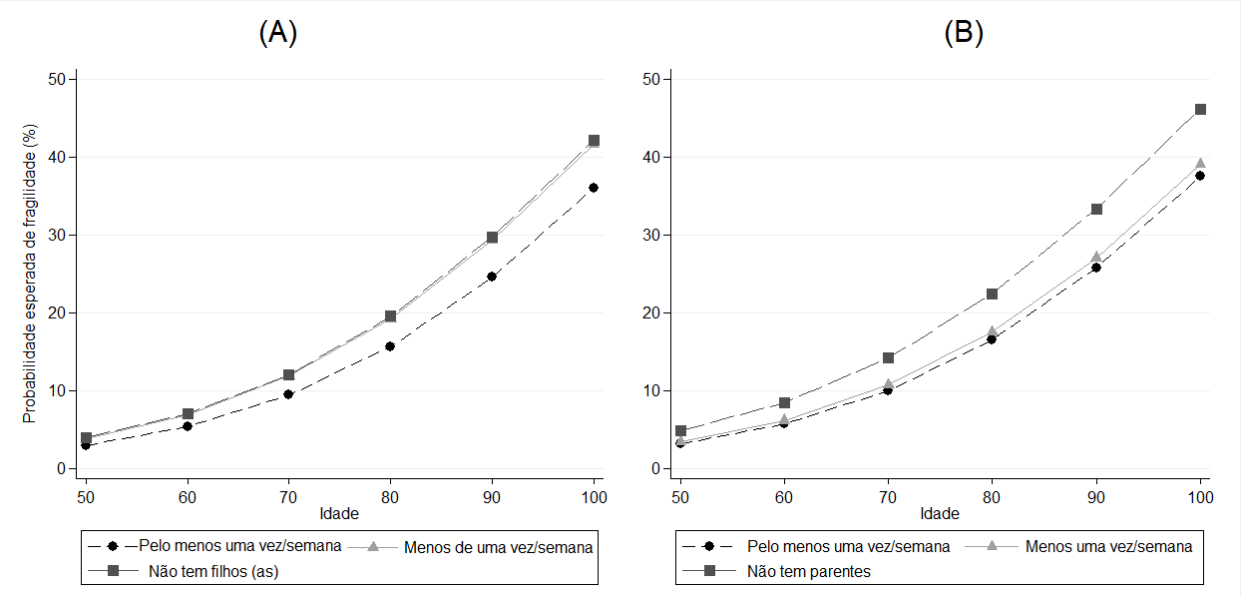
Probabilidade esperada de fragilidade em função da idade e frequência de contato virtual com filhos(as) (A) e parentes (B) (ELSI-Brasil, 2015–2016).

 De acordo com a Figura 1 , a probabilidade esperada de fragilidade aumenta à medida que a idade em todos os grupos de frequência de contato virtual também aumenta. Entretanto, nota-se, na Figura 1 (A), que a probabilidade é menor no grupo de maior frequência de contato virtual com filhos(as), chegando 36,1% dos indivíduos desse grupo aos 100 anos; na frequência de contato virtual menor, em torno de 42% dos indivíduos na mesma idade. Padrão semelhante é visto na Figura 1 (B), para frequência de contato virtual com parentes, com probabilidade de fragilidade maior entre o grupo que não tem parentes (46,2%) aos 100 anos. 

## DISCUSSÃO

Este estudo mostrou que os elementos da rede social que foram consistentemente associados à pré-fragilidade e fragilidade foram a característica dos laços da rede social (frequência baixa de contato virtual com filhos(as) e não ter parentes), o apoio social (indisponibilidade de ajuda com empréstimos) e a interação social negativa (sentir solidão). Outros elementos foram associados somente à fragilidade: percepção de críticas e não ter filhos.

 Neste estudo, a pré-fragilidade e fragilidade foram associadas positivamente a não ter parentes e à frequência não semanal de contatos virtuais com filhos(as). Ainda que adultos mais velhos possam ter dificuldade em utilizar equipamentos que permitam o contato virtual, é possível que filhos tenham maior facilidade para o contato virtual em detrimento do presencial em função de suas rotinas com seus próprios filhos, trabalhos e estudos ^21^ . Entre indivíduos coreanos na faixa etária de 70-84 anos, houve associação positiva entre frequência de contato mensal ou raro com amigos e pré-fragilidade e fragilidade ^10^ . No Brasil, resultados semelhantes foram encontrados para menos que três encontros mensais com amigos e incapacidade ^22^ . Entretanto, nenhum dos estudos mencionados diferenciou os contatos do tipo virtual dos contatos presenciais. 

 Em relação à associação negativa do apoio social instrumental com a pré-fragilidade e fragilidade, os achados desta pesquisa foram divergentes daqueles reportados no município de Natal (RN) ^23^ e Ribeirão Preto (SP) ^11^ , em que o apoio social não se associou à fragilidade. Contar com apoio social instrumental, principalmente no que se refere às questões financeiras, pode ser um fator de despreocupação com o futuro, uma vez que a pressão financeira atua como um estressor crônico e aumenta o risco para fragilidade ^7^ . 

 Mais de 90% dos participantes relataram disponibilidade para receber apoio social instrumental e emocional, exceto pré-frágeis e frágeis em relação ao apoio social instrumental (empréstimos de dinheiro e/ou objetos). Estudos desenvolvidos em cidades brasileiras também evidenciaram elevada disponibilidade de apoio social, variando de 91,4% em Belo Horizonte (MG) ^22^ a 98,8% em Ivoti (RS) ^12^ . 

 Porém, a ideia de que o apoio social estará disponível em caso de necessidade pode ou não corresponder à prestação efetiva de apoio quando necessário ^6^ . Nesse sentido, contar somente com o apoio social informal prestado por amigos, vizinhos e familiares pode não ser a opção mais adequada ^24^ . Um estudo longitudinal conduzido com holandeses de 65 anos ou mais não encontrou diminuição do apoio social emocional e instrumental prestado entre os frágeis durante o acompanhamento de três anos ^25^ . No entanto, um estudo longitudinal realizado em Ribeirão Preto (SP) evidenciou que, ao longo de 10 anos, houve uma redução na média do apoio social prestado pela família, amigos e serviço de saúde aos adultos mais velhos ^11^ . 

 Considerando-se a interação social negativa, a solidão associou-se positivamente à pré-fragilidade e à fragilidade, enquanto a percepção de críticas pelo indivíduo se associou somente à fragilidade. Aqueles que reportaram solidão apresentaram chance 36% e 40% maior de pré-fragilidade e fragilidade, respectivamente. A associação entre solidão e pré-fragilidade e fragilidade também foi encontrada em estudos longitudinais, de forma bidirecional: solidão como fator de risco para a fragilidade ^3^^,^^4^ e fragilidade como fator de risco para a solidão ^26^ . Diferentes estudos longitudinais internacionais apontam a rarefação de contatos sociais entre pessoas de 65 anos ou mais. Um estudo com holandeses verificou o aumento da solidão entre os frágeis, ao longo de três anos ^25^ ; em outra pesquisa, com mexicanos que vivem nos Estados Unidos, 16% relataram que “quase nunca” tinham alguém para conversar, contar ou falar de seus problemas ^7^ . Um estudo transversal na Coréia, aponta que 11 a 15% dos indivíduos não tinham com quem conversar e contar ^27^ . 

 A necessidade de se conectar é uma característica humana e está diretamente associada aos vínculos e aos sentimentos de companheirismo ^28^ , de modo que o fato de não ter filhos, parentes ou amigos pode gerar sentimentos de solidão. É possível, inclusive, que uma das causas de interações sociais negativas persistentes vivenciadas por adultos mais velhos seja o descompasso entre a necessidade de apoio social por eles requerida e a capacidade de oferta pelos membros da rede social, o que produziria tensões ^1^ . Assim, avaliar o sentimento de solidão e a qualidade das relações estabelecidas pelos adultos mais velhos precisa ser uma das prioridades dos serviços de saúde e assistência social diante dos riscos para a fragilidade e futuras incapacidades ^4^ . 

 Segundo o modelo conceitual de Berkman e Krishna ^6^ , o Estado faz parte da estrutura macrossocial da rede social dos indivíduos. Assim, é papel dele promover ações que ampliem o apoio social, o cuidado e o amparo a esses indivíduos e suas famílias ^24^ . Neumann e Albert ^29^ ressaltam a urgência de o Brasil ofertar políticas de cuidado de longa duração, em razão do número crescente de adultos mais velhos com incapacidades e a diminuição da disponibilidade de cuidado pela família. Os autores salientam a necessidade de o Estado reconhecer o valor do cuidado prestado pelas famílias, devendo proporcionar a elas, inclusive, apoio financeiro. 

 Uma experiência exitosa de apoio social instrumental e emocional é o Programa Maior Cuidado em Belo Horizonte (MG), que apoia famílias vulneráveis no cuidado domiciliar a pessoas idosas que precisam de ajuda para executar atividades básicas da vida diária, prevenindo situações de risco, exclusão, isolamento social e sobrecarga familiar, de forma intersetorial ^30^ . 

 A intervenção na rede social dos indivíduos pode atuar como um fator modificador da fragilidade, além das intervenções já descritas na literatura como: atividade física, suplementação proteica/calórica em casos de desnutrição e perda de peso, manejo da sarcopenia ^2^ . A implementação pelo poder público de Grupos de Convivência, Centros-Dia, Casas-lar, Repúblicas e programas como o Maior Cuidado são intervenções possíveis na rede social dos indivíduos, as quais oportunizam o apoio social com impacto na saúde e bem-estar dos indivíduos e de suas famílias. 

Este estudo apresenta pontos fortes e fracos. Como ponto forte, destaca-se o pioneirismo do estudo ao analisar vários elementos da rede social em uma amostra representativa nacional. Contudo, seu caráter transversal não permite estabelecer relações de causalidade entre rede social e fragilidade. Além disso, todas as variáveis de rede social incluídas refletem a percepção dos indivíduos, o que nem sempre traduz a realidade. Além disso, as questões de apoio social instrumental incluídas refletiam uma expectativa de receber ajuda de outras pessoas em caso de uma eventual necessidade de saúde, o que pode não condizer com a real disponibilidade de ajuda. Outra limitação foi a ausência de informações referentes ao número de amigos e vizinhos para a mensuração do tamanho da rede social, o que pode ter explicado a maior média do tamanho da rede social entre os indivíduos frágeis, em comparação aos não frágeis e pré frágeis.

Desse modo, estudos futuros poderiam mensurar objetivamente a disponibilidade da oferta de apoio pelos membros da rede social ou fazer a contagem dos membros da rede social em relação a laços fortes ou fracos de provimento de apoio social. Ressalta-se que o ELSI-Brasil é um estudo de coorte prospectiva, e futuras análises dos mesmos elementos da rede social em perspectiva longitudinal poderão esclarecer a temporalidade das associações encontradas.

Por fim, reforça-se a fragilidade em sua perspectiva multidimensional, de modo que intervenções na rede social têm o potencial para diminuir e prevenir a fragilidade. Formuladores de políticas públicas, autoridades e profissionais de saúde e assistência social devem abranger a rede social de adultos mais velhos, em relação à característica dos laços da rede social, ao apoio social e à interação social negativa. Implementar os serviços já garantidos em lei precisa ser uma ação imediata do Estado como forma de apoiar a família no cuidado, ampliar as possibilidades de apoio social prestado e reduzir sentimentos de solidão.
