# Supplementary material for: Fragilidade e rede social entre adultos brasileiros mais velhos: evidências do ELSI-Brasil
Source: Rev Saude Publica. 2024 Dec 16;58:51. [Article in Portuguese] doi: 10.11606/s1518-8787.2024058005525 (PMC11655055; doi:10.11606/s1518-8787.2024058005525)
Supplement: Translation

# Translation: Frailty and social network among older Brazilian adults: evidence from ELSI-Brazil

**Published:** 2024-12-16

**Authors:** Karla Geovani Silva Marcelino, Luciana de Souza Braga, Fabiola Bof de Andrade, Karla Cristina Giacomin, Maria Fernanda Lima-Costa, Juliana Lustosa Torres

**Affiliations:** I Universidade Federal de Minas Gerais. Faculdade de Medicina. Programa de Pós-Graduação em Saúde Pública. Belo Horizonte, MG, Brasil; II Universidade Federal de Minas Gerais. Faculdade de Medicina. Departamento de Medicina Preventiva e Social. Belo Horizonte, MG, Brasil; III Fundação Oswaldo Cruz. Instituto René Rachou. Saúde Coletiva. Belo Horizonte, MG, Brasil; IV Prefeitura Municipal de Belo Horizonte. Secretaria Municipal de Saúde. Belo Horizonte, MG, Brasil

**Keywords:** Social Relations, Social Support, Frailty, Aging

## Abstract

To investigate the elements of the social network associated with frailty syndrome in older Brazilian adults.

Baseline data from the Brazilian Longitudinal Study of Aging (ELSI-Brazil, 2015–2016) were used. Frailty was defined by the Fried phenotype (unintentional weight loss, exhaustion, weakness, slowness, and low level of physical activity). The social network was assessed using the conceptual model of Berkman and Krishna (social network structure, characteristics of social network ties, social support, and negative social interaction). Potential confounding variables included sociodemographic (age, sex, education, self-reported race, *per capita* family income, and place of residence) and health characteristics (polypharmacy, multimorbidity, depression, falls, hospitalization, and cognitive function). Analyses were based on multinomial logistic regression.

Among the 8,629 participants, 53.5% were pre-frail individuals and 9.1% were frail individuals. The elements of the social network that were consistently associated with pre-frailty and frailty were the following: characteristics of social network ties, social support, and negative social interaction. A positive association was found for less-than-weekly frequency of virtual contact with sons and daughters (OR = 1.15; 95%CI 1.01–1.33 for pre-frailty and OR = 1.51; 95%CI 1.13–2.02 for frailty) and for loneliness (OR = 1.36; 95%CI 1.19–1.56 for pre-frailty and OR = 1.40; 95%CI 1.12–1.75 for frailty). A negative association was found for social support (help with loans) (OR = 0.75; 95%CI 0.60–0.94 for pre-frailty and OR = 0.54; 95%CI 0.40–0.74 for frailty). However, the perception of criticism was only associated with frailty (OR = 1.35; 95%CI 1.11–1.64).

Social network is an important element for reducing/preventing frailty in older adults. Therefore, public policies and health and social assistance professionals should encompass the older adults’ social network regarding the characteristics of social network ties, social support, and negative social interaction.

## INTRODUCTION

 Frailty is a major public health challenge due to its negative impact on individuals’ functionality, which can lead to the need for long-term care and family (re)organization ^1^^,^^2^ . This syndrome is characterized by increased vulnerability to low homeostatic and adaptive response to minor stressful events ^3^^,^^4^ , and often goes unnoticed by health professionals. From a multidimensional perspective, frailty is influenced by socio-familial vulnerability, whether due to the lack of social support, defined as limited availability of help, or by a scarce social network ^3^^,^^5^ , which constitutes a scarce tangle of social relationships established by the individual ^6^ . 

 Berkman and Krishna ^6^ , in line with theorists such as Durkheim and Bowlby, proposed a conceptual model on the impact of the social network on health, based on a macrosocial structure. This model establishes that social networks are embedded in a broader socioeconomic, political, and cultural context that determines the structure itself (size, density, and distance, among others) and the characteristics of the social network ties (frequency of in-person or virtual contacts, reciprocity, and intimacy, among others), providing psychosocial mechanisms that can impact health. Among the mechanisms, social support (positive impact) and negative social interactions (negative impact) stand out. 

 At an international level, longitudinal data from Mexicans aged 65 or over living in the United States showed that increased emotional social support, defined as demonstrations of care and affection for others, was associated with a lower progression of frailty among those who were moderately frail ^7^ . 

 Longitudinal and cross-sectional studies reported a positive association between the absence of a partner ^8^^,^^9^ and loneliness ^3^ and pre-frailty and frailty. Regarding the characteristics of social network ties, the literature indicates that a monthly or lower frequency of contact with friends ^10^ , as well as a social network with few exchanges, are associated with a higher prevalence of frailty ^5^ . A Brazilian longitudinal study, conducted with people aged 65 or over, found no association between social support and frailty ^11^ , but this association was verified in a cross-sectional study ^12^ . Nevertheless, the Brazilian studies were not based on a nationally representative sample and have not evaluated the social network. 

Several studies have used the term “social network” as a synonym for social support, without distinguishing the possible mechanisms of association with health. Thus, this study is based on the assumption that the social network (social network structure, characteristics of social network ties, and social support) can act as a protective or risk factor (negative social interaction) for frailty. Therefore, the objective of this study is to investigate the elements of the social network associated with frailty in older Brazilian adults.

## METHODS

### Study design

 This is a cross-sectional study, which used baseline data from the Brazilian Longitudinal Study of Aging (ELSI-Brazil, 2015–2016), performed in 2015–2016. ELSI-Brazil is a cohort study conducted on a representative sample of the Brazilian population aged ≥ 50 years. All residents aged 50 years or older in the selected households were eligible to participate, totaling 9,412 participants. More details on the sample selection process can be found in a previous publication ^13^ . ELSI-Brazil was approved by the Ethics Committee of the Fundação Oswaldo Cruz, Minas Gerais, Brazil (number: 34649814.3.0000.5091). All participants signed the Informed Consent Form. 

### Dependent variable

 Frailty was defined by the phenotype of Fried et al. ^14^ , according to the number of positive criteria: three or more as “frail,” one or two as “pre-frail,” and none as “non-frail.” The criteria were: (1) Weight loss: self-reported weight loss of 4.5 kg or more in the last three months, without any intention/diet; (2) Exhaustion: frequencies greater than 3-4 days for any of the following questions from the Center for Epidemiological Studies Depression Scale (CES-D) ^15^ . “Did you feel that you couldn’t get going?”; “Did you feel that everything you do is an effort?”; (3) Weakness: handgrip strength in the lowest quintile, after adjusting for sex and body mass index (BMI) quartiles, and those who were bedridden or unable to perform the test ^16^ . Strength was assessed in three attempts, using a hand dynamometer on the dominant upper limb and considering the best performance; (4) Slowness: quintile of longest time spent walking 3 m in the usual way, stratified by sex and height, and those who were bedridden or unable to perform the test ^16^ ; and (5) Low physical activity: lowest quintile of energy expenditure measured in kilocalories (kcals) per week, stratified by sex ^16^ . The kcals spent in the last week on activities carried out at work, going from one place to another, leisure, sports, practicing exercise or performing household chores were counted considering the intensity (light, moderate, and vigorous) and time (minutes/hours), based on the Short Form of the International Physical Activity Questionnaire (IPAQ) ^17^ . 

### Independent variables

 The independent variables were those related to the elements of the social network, based on the conceptual model of Berkman and Krishna ^6^ on the impact of the social network on health. The elements selected for this study were: social network structure, characteristics of the social network ties, social support, and negative social interaction. 

### Social network structure

 According to the conceptual model of Berkman and Krishna ^6^ , the social network structure can be assessed by size, density, and distance, among others. This study focused on the social network size and the type of family living arrangement. 

The social network size was assessed by adding the total number of people who are part of the participant’s social network, considering children, grandchildren or great-grandchildren, and living siblings, based on three questions about the number of (1) living children, (2) living grandchildren or great-grandchildren, and (3) living siblings.

Friends and/or neighbors were not included in the social network size, since the number of people for the ties in question is not included in the ELSI-Brazil baseline. The total social network size was used as a continuous variable and, due to its distribution, was truncated to 50 people. The family living arrangement was assessed by self-report, considering living alone, living with a partner, or other arrangements.

### Characteristics of social network ties

 According to the conceptual model of Berkman and Krishna ^6^ , the characteristics of social network ties can be assessed based on the frequency of contacts (in-person or virtual), reciprocity, multiplicity, duration, and intimacy. This study focused on the frequency of contacts. 

The frequency of contacts with the social network was defined separately for in-person and virtual contacts, considering only individuals who did not live with the participant.

“In-person contact” was defined as personal encounters, and “virtual contact” was defined as conversations by telephone, Skype, WhatsApp, Facebook, with children, relatives, or friends. The frequency of contacts was assessed by self-report, according to three response categories: at least once/week, less than once/week, or no link related to the category analyzed (children, relatives, or friends).

### Social support

Instrumental social support was assessed by self-report on the availability of help: with the house (yes or no); with going shopping, paying bills, or going to the bank (yes or no), in case of illness; and with loans, including money or objects (yes or no). Emotional social support was assessed by self-report on the availability of a person to confide in (yes or no).

### Negative social interaction

Assessed based on the individual’s perception of loneliness, criticism, and excessive care. Loneliness, assessed based on the perception of feeling alone or lonely, by a single question: “How often do you feel alone (lonely)?” (hardly ever, some of the time, often). Perception of criticism assessed by the question: “Do you think that people make too many demands from or criticisms of you?” (never, sometimes, or always). Perception of excessive care, by the question: “Do you feel bothered because you think that people try to help you more than you think you need?” (never, sometimes, or always) For the three variables, the response categories “sometimes” and “always” were grouped together.

### Potential confounding variables

 Sociodemographic and health-related characteristics were considered potential confounding variables. The sociodemographic variables were: age (50–59; 60–69; 70–79; ≥ 80 years); sex (female or male); education, in completed years (never studied, 1–4 years, 5–8 years, ≥ 9 years); self-reported race (White, Black, Mixed-race or other); *per capita* family income, in tertiles [lower up to R$ 558.70), middle (from R$ 558.71 to R$ 1,000.00) and upper (≥ R$ 1,000.01)] tertiles; and place of residence (urban or rural). The health-related characteristics were: health-perception (very good/good, fair, or poor/very poor); polypharmacy, considering the regular use of five or more medications prescribed by a doctor ^18^ and used in the last two weeks (yes or no); multimorbidity ^19^ (yes or no); medical diagnosis of depression (yes or no); self-report on falls in the last 12 months (yes or no); hospitalization in the last 12 months, considering hospitalizations for at least 24 hours (yes or no), and cognitive function. 

 Multimorbidity was considered the presence of two or more chronic conditions, including cardiovascular diseases (arterial hypertension, stroke, acute myocardial infarction, angina, and heart failure), chronic kidney disease, chronic neurological disease (Alzheimer’s disease and Parkinson’s disease), chronic respiratory disease (emphysema, chronic obstructive pulmonary disease, and bronchitis), diabetes, arthritis, asthma, cancer, and obesity. All chronic conditions were obtained by self-report on a history of medical diagnosis, except obesity, which was characterized based on the objective measurement of weight and height from the BMI calculation (≥ 30 kg/m2 for those under 60 years old, and ≥ 27 kg/m2 for those aged 60 or over) ^19^ . Cognitive function was assessed based on language and executive function, using the one-minute semantic verbal fluency test, considering the total number of animals mentioned ^20^ . 

### Statistical analysis

Frequency distributions were calculated for categorical variables, observing differences using Pearson’s chi-square test, with Rao-Scott correction. For continuous variables, the mean and 95% confidence interval (95%CI) were calculated. Differences between frailty categories were assessed using the adjusted Wald test.

For group and adjusted analyses, multinomial logistic regression models were used to estimate odds ratios (OR) and their respective 95%CI of the association between social network and frailty. The analyses were performed separately by blocks of social network variables and, subsequently, adjusted for all potential confounding variables, as follows: (1) social network structure; (2) characteristics of social network ties (in-person contact); (3) characteristics of social network ties (virtual contact); (4) social support; and (5) negative social interaction. The multicollinearity test was used to test the correlation between the variables included in the multivariate models. The variables related to the characteristics of social network ties (frequency of contacts) associated with frailty were plotted in a figure. All analyses were performed using Stata/SE® software (StataCorp., CollegeStation, United States), version 14.2, considering the sample design and participants’ weights.

## RESULTS

 With regard to the 9,412 participants in the ELSI-Brazil baseline, 8,629 (91.7%) had complete information about frailty classification and were included in the study. The mean age was 62.2 years (95%CI 61.4–63.1). Among the participants, 53.5% (95%CI 51.8–55.1) were pre-frail individuals and 9.1% (95%CI 8.0–10.2) were frail individuals. Participants’ characteristics according to frailty are described in Table 1 . Among the frail individuals, 50.8% were under 70 years of age, 44.8% had up to four years of schooling, and 34.0% had poor/very poor self-perceived health. All characteristics showed significant differences between the frailty categories, except for place of residence. 

**Table 1.** Distribution of sociodemographic and health-related characteristics, total and according to frailty classification (ELSI-Brazil, 2015–2016).

**CHARACTERISTIC**	**FRAILTY CLASSIFICATION**				
	**Total**	**Non-frail**	**Pre-frail**	**Frail**	**p-value**
**SOCIODEMOGRAPHIC**					
**Age (%)**					< 0.001
50–59 years	48.3	55.7	47.3	23.7	
60–69 years	29.9	31.0	29.6	27.1	
70–79 years	15.4	11.3	16.3	26.6	
≥ 80 years	6.4	2.0	6.8	22.6	
**Female (%)**	53.7	50.8	55.3	56.2	0.007
**Schooling (%)**					< 0.001
Never studied	12.4	7.5	13.5	26.0	
1–4 years	38.4	33.2	41.0	44.8	
5–8 years	21.9	23.7	21.1	19.0	
≥ 9 years	27.3	35.6	24.4	10.2	
**Self-reported race (%)**					0.045
White	42.8	45.1	41.6	40.4	
Black	9.6	8.3	10.2	11.6	
Mixed-race	44.7	44.0	45.3	43.8	
Other	2.9	2.6	2.9	4.2	
**Per capita family income (%)**					< 0.001
Lower tertile	31.6	26.8	33.7	39.0	
Middle tertile	32.9	32.0	33.0	36.4	
Upper tertile	35.5	41.2	33.3	24.6	
**Place of urban residence (%)**	84.8	85.9	84.2	83.6	0.399
**HEALTH-RELATED**					
**Health self-perception (%)**					< 0.001
Very good/good	43.8	56.3	38.9	20.6	
Fair	44.8	39.4	48.5	45.4	
Poor/very poor	11.4	4.3	12.6	34.0	
**Polypharmacy** ^a^ **(%)**	13.1	7.5	14.7	26.9	< 0.001
**Multimorbidity** ^b^ **(%)**	55.9	48.6	59.3	67.0	< 0.001
**Depression (%)**	18.6	12.4	20.6	32.3	< 0.001
**Fall in the last 12 months (%)**	21.8	15.7	23.6	37.0	< 0.001
**Average cognitive function (95%CI)**	11.8 (11.5–12.1)	13.1 (12.8–13.5)	11.5 (11.2–11.8)	8.2 (7.7–8.8)	< 0.001
**Hospitalization in the last 12 months (%)**	9.8	5.6	10.7	21.9	< 0.001
**Total n**	8,629	3,117	4,655	857	

95%CI: 95% confidence interval.

a Use of five or more medications on a regular basis.

b Two or more chronic conditions, including cardiovascular disease (high blood pressure, stroke, acute myocardial infarction, angina, and heart failure), chronic kidney disease, chronic neurological disease (Alzheimer’s disease and Parkinson’s disease), chronic respiratory disease (emphysema, chronic obstructive pulmonary disease, and bronchitis), diabetes, arthritis, asthma, cancer, and obesity.

**p-value:** Pearson’s chi-square test with Rao-Scott correction for categorical variables and adjusted Wald test for continuous variables.

 The distribution of social network elements according to frailty is described in Table 2 . The participants had a social network of 13 people on average (95%CI 12.4–13.8), and approximately 65% ​​lived with a partner. Among the frail individuals, the highlights are the largest average social network size (16.1; 95%CI 14.8–17.4) and the highest level of frequency of contacts, less than once/week, in-person (67.9%) and virtual (63.0%) with relatives. It is also possible to observe that, in this group, there is lower prevalence of instrumental social support (availability of help with loans) and emotional support (availability of someone to confide in), and higher prevalence of loneliness (58.8%) and perception of excessive care (39%). 

**Table 2.** Distribution of the structure and characteristics of social network ties, social support, and negative social interaction, total and according to frailty classification (ELSI-Brazil, 2015–2016).

**SOCIAL NETWORK CARACTERISTIC**	**TOTAL**	**Frailty CLASSIFICATION**			
		**Non-frail**	**Pre-frail**	**Frail**	**p-value**
**SOCIAL NETWORK STRUCTURE**					
**Social network size, mean (95%CI)**	13.0(12.4–13.5)	11.8 (11.2–12.4)	13.2(12.7–13.7)	16.1(14.8–17.4)	< 0.001
**Family living arrangement (%)**					< 0.001
Living alone	8.6	7.4	9.2	9.6	
Living with partner	64.7	69.5	62.9	56.1	
Other arrangements	26.7	23.1	27.9	34.3	
**CHARACTERISTICS OF SOCIAL NETWORK TIES**					
**Frequency of in-person contact with children**					< 0.001
At least once/week	48.3	47.7	48.5	49.7	
Less than once/week	28.5	26.2	29.4	32.3	
No children	23.2	26.1	22.1	18.0	
**Frequency of in-person contact with relatives**					< 0.001
At least once/week	34.6	38.9	33.1	25.3	
Less than once/week	62.0	59.1	63.2	67.9	
No relatives	3.4	2.0	3.7	6.8	
**Frequency of in-person contact with friends**					< 0.001
At least once/week	69.7	72.9	68.8	62.0	
Less than once/week	20.1	19.7	19.9	23.1	
No friends	10.2	7.4	11.3	14.9	
**Frequency of virtual contact with children**					< 0.001
At least once/week	56.7	58.3	57.0	48.0	
Less than once/week	20.0	15.6	20.8	33.9	
No children	23.3	26.1	22.2	18.1	
**Frequency of virtual contact with relatives**					< 0.001
At least once/week	45.8	53.2	43.3	30.2	
Less than once/week	50.8	44.8	52.9	63.0	
No relatives	3.4	2.0	3.8	6.8	
**Frequency of virtual contact with friends**					< 0.001
At least once/week	43.4	49.9	41.8	25.6	
Less than once/week	46.3	42.7	46.8	59.1	
No friends	10.3	7.4	11.4	15.3	
**SOCIAL SUPPORT**					
INSTRUMENTAL SOCIAL SUPPORT					
Availability of help with the house (%)	97.4	98.3	96.7	97.0	< 0.001
Availability of help with going shopping, paying bills, or going to the bank (%)	98.4	99.0	97.9	98.3	0.004
Availability of help with loans (%)	90.2	92.8	89.0	84.8	< 0.001
EMOTIONAL SOCIAL SUPPORT					
Availability of person for confidences (%)	92.3	93.4	91.5	91.2	0.046
**NEGATIVE SOCIAL INTERACTION**					
Loneliness some of the time/often (%)	47.2	39.5	51.2	58.8	< 0.001
Perception of criticism sometimes/always (%)	47.0	45.4	48.1	47.6	0.111
Perception of excessive care sometimes/always (%)	32.0	29.7	32.7	39.0	0.001
**Notal n**	8,629	3,117	4,655	857	

95%CI: 95 95% confidence interval.

**P-value:** Pearson’s chi-square test with Rao-Scott correction for categorical variables and adjusted Wald test for continuous variables.

 The results of the multinomial logistic regression are described in Table 3 . As multicollinearity was not evidenced (Variance Inflation Factor [VIF] < 2), all confounding variables were maintained in the adjusted models. Considering the adjusted models, the chance of pre-frailty was higher among those who reported frequency of virtual contact with children (OR = 1.15; 95%CI 1.01–1.33) and relatives (OR = 1.18; 95%CI 1.05–1.34) less than once/week, had no relatives or friends, and reported feeling lonely some of the time/often (OR = 1.36; 95%CI 1.19–1.56). We also observed that the chance of pre-frailty was lower among those who reported availability of help with the house (OR = 0.66; 95%CI 0.46-0.94) and with loans (OR = 0.75; 95%CI 0.60–0.94). 

**Table 3.** Results of the models per group and adjusted for the association between the structure and characteristics of social network ties, social support, and negative social interaction, total and according to frailty classification (ELSI-Brazil, 2015–2016).

**CHARACTERISTIC**	**Models per group**		**Adjusted models**	
	**Pre-frail** **OR (95%CI)**	**Frail** **OR (95%CI)**	**Pre-frail** **OR (95%CI)**	**Frail** **OR (95%CI)**
**SOCIAL NETWORK STRUCTURE** ^a^				
**Social network size**	1.02 (1.00–1.02)	1.05* (1.04–1.06)	1.00 (0.99–1.00)	0.99 (0.98–1.00)
**Family living arrangement (versus living alone)**				
Living with partner	0.75* (0.64–0.86)	0.65* (0.48–0.86)	0.87 (0.75–1.01)	0.90 (0.65–1.25)
Other arrangements	1.00 (0.83–1.22)	1.09 (0.76–1.58)	1.06 (0.85–1.32)	1.03 (0.67–1.58)
**CHARACTERISTICS OF SOCIAL NETWORK TIES** ^b,c^				
**Frequency of in-person contact with children (versus at least once/week)**				
Less than once/week	1.07 (0.94–1.22)	1.09 (0.85–1.41)	1.04 (0.90–1.21)	0.93 (0.72–1.19)
No children	0.84* (0.71–0.99)	0.67* (0.49–0.91)	1.01 (0.85–1.20)	1.15 (0.81–1.63)
**Frequency of in-person contact with relatives (versus at least once/week)**				
Less than once/week	1.19* (1.07–1.33)	1.58* (1.28–1.95)	1.10 (0.97–1.25)	1.16 (0.93–1.43)
No relatives	2.14* (1.55–2.95)	4.99* (2.92–8.51)	1.73* (1.21–2.48)	2.73* (1.46–5.10)
**Frequency of in-person contact with friends (versus at least once/week)**				
Less than once/week	1.06 (0.91-1.22)	1.36* (1.03–1.80)	1.00 (0.84–1.18)	1.05 (0.78–1.42)
No friends	1.55* (1.30–1.86)	2.20* (1.62–3.00)	1.24* (1.04–1.48)	1.35 (0.92–1.98)
**Frequency of virtual contact with children (versus at least once/week)**				
Less than once/week	1.20* (1.04–1.39)	1.87* (1.48–2.36)	1.15* (1.01–1.33)	1.51* (1.13–2.02)
No children	0.89 (0.76–1.04)	0.89 (0.67–1.18)	1.04 (0.89–1.22)	1.40* (1.02–1.93)
**Frequency of virtual contact with relatives (versus at least once/week)**				
Less than once/week	1.32* (1.19–1.46)	1.71* (1.36–2.16)	1.18* (1.05–1.34)	1.25 (0.95–1.63)
No relatives	2.11* (1.49–2.98)	4.11* (2.43–6.96)	1.77* (1.22–2.56)	2.49* (1.41–4.41)
**Frequency of virtual contact with friends (versus at least once/week)**				
Less than once/week	1.15* (1.00–1.32)	1.98* (1.58–2.50)	0.98 (0.84–1.15)	1.12 (0.84–1.49)
No friends	1.62* (1.35–1.96)	2.95* (2.12–4.10)	1.23* (1.01–1.50)	1.36 (0.90–2.05)
**SOCIAL SUPPORT** ^d^				
INSTRUMENTAL SOCIAL SUPPORT				
Availability of help with the house (%) ( *versus* no help)	0.64* (0.45–0.91)	0.68 (0.38–1.22)	0.66* (0.46–0.94)	0.67 (0.29–1.53)
Availability of help with going shopping, paying bills, or going to the bank ( *versus* no help)	0.63 (0.37–1.05)	0.92 (0.42–2.03)	0.62 (0.35–1.10)	1.13 (0.46–2.74)
Availability of help with loans ( *versus* no help)	0.68* (0.56–0.83)	0.44* (0.34–0.57)	0.75* (0.60–0.94)	0.54* (0.40–0.74)
EMOTIONAL SOCIAL SUPPORT				
Availability of person for confidences ( *versus* no availability)	0.89 (0.71–1.11)	0.88 (0.57–1.36)	0.95 (0.73–1.20)	0.93 (0.60–1.46)
**NEGATIVE SOCIAL INTERACTION** ^e^				
Loneliness some of the time/often ( *versus* hardly ever)	1.60* (1.41–1.82)	2.12* (1.74–2.59)	1.36* (1.19–1.56)	1.40* (1.12–1.75)
Perception of criticism sometimes/always ( *versus* never)	0.98 (0.88–1.09)	0.89 (0.74–1.06)	1.09 (0.98–1.23)	1.35* (1.11–1.64)
Perception of excessive care sometimes/always ( *versus* never)	1.07 (0.95–1.20)	1.39* (1.11–1.74)	0.94 (0.83–1.06)	0.93 (0.70–1.23)

OR: odds ratio.

95CI: 95% confidence interval. Models based on multinomial logistic regression, with non-frail reference category. Models adjusted for age, sex, education, self-reported race, per capita family income, place of residence, health self-perception, polypharmacy, multimorbidity, depression, falls, cognitive function, hospitalization.

a Final n = 7,802.

b Final n = 8,002.

c Final n = 7,902.

d Final n = 7,086.

e Final n = 7,220.

* p < 0.05.

The chance of frailty was higher among those who reported frequency of virtual contact with children (OR = 1.51; 95%CI 1.13–2.02) less than once/week, had no children or relatives, reported loneliness (OR = 1.40; 95%CI 1.12–1.75) and perception of criticism (OR = 1.35; 95%CI 1.11–1.64) sometimes/always. On the other hand, lower odds of frailty were found for availability of help with loans (OR = 0.54; 95%CI 0.40–0.74).

 According to Figure 1 , the predicted probability of frailty increases as age increases in all groups of frequency of virtual contacts. However, it can be seen in Figure 1 (A) that the probability is lower in the group with the highest frequency of virtual contacts with children, reaching 36.1% of individuals in this group at 100 years of age; in the lowest frequency of virtual contacts, it reaches around 42% of individuals at the same age. A similar pattern is seen in Figure 1 (B), for frequency of virtual contacts with relatives, with a higher probability of frailty among the group that has no relatives (46.2%) at 100 years of age. 

**Figure 1.** Predicted probability of frailty according to age and frequency of virtual contact with children (A) and relatives (B) (ELSI-Brazil, 2015–2016).

## DISCUSSION

This study showed that the elements of the social network that were consistently associated with pre-frailty and frailty were the characteristics of social network ties (low frequency of virtual contacts with children and no relatives), social support (unavailability of help with loans), and negative social interaction (feeling lonely). Other elements were associated only with frailty: perception of criticism and no children.

 In this study, pre-frailty and frailty were positively associated with not having relatives and the non-weekly frequency of virtual contact with children. Although older adults may have difficulty using equipment that allows virtual contact, it is possible that sons and daughters have greater ease with virtual contact than in-person contact due to their routines with their own children, work, and studies ^21^ . Among Korean individuals in the 70-84 age group, there was a positive association between the frequency of monthly or rare contact with friends and pre-frailty and frailty ^10^ . In Brazil, similar results were found for less than three monthly meetings with friends and disability ^22^ . However, none of the mentioned studies differentiated virtual contacts from in-person contacts. 

 Regarding the negative association of instrumental social support with pre-frailty and frailty, the findings of this study diverged from those reported in the cities of Natal (state of Rio Grande do Norte) ^23^ and Ribeirão Preto (state of São Paulo) ^11^ , where social support was not associated with frailty. Relying on instrumental social support, especially regarding financial issues, can be a factor for the older adult not worrying about the future, since financial pressure acts as a chronic stressor and increases the risk of frailty ^7^ . 

 More than 90% of participants reported availability of instrumental and emotional social support, except for pre-frail and frail individuals in relation to instrumental social support (loans of money and/or objects). Studies conducted in Brazilian cities have also shown a high level of availability of social support, ranging from 91.4% in Belo Horizonte (state of Minas Gerais) ^22^ to 98.8% in Ivoti (state of Rio Grande do Sul) ^12^ . 

 However, the idea that social support will be available in case of need may or may not correspond to the actual provision of support when needed ^6^ . In this sense, relying solely on informal social support provided by friends, neighbors, and family may not be the most appropriate option ^24^ . A longitudinal study conducted with Dutch individuals aged 65 or over found no decrease in emotional and instrumental social support provided among the frail during a three-year follow-up ^25^ . Nevertheless, a longitudinal study conducted in Ribeirão Preto (state of São Paulo) showed that, over the course of 10 years, there was a reduction in the average amount of social support provided by family, friends, and health services to older adults ^11^ . 

 Considering negative social interaction, loneliness was positively associated with pre-frailty and frailty, while the individual’s perception of criticism was only associated with frailty. Those who reported loneliness had 36% and 40% greater chance of pre-frailty and frailty, respectively. The association between loneliness and pre-frailty and frailty was also found in longitudinal studies, in a bidirectional manner: loneliness as a risk factor for frailty ^3^^,^^4^ and frailty as a risk factor for loneliness ^26^ . Various international longitudinal studies point to the scarcity of social contacts among people aged 65 or over. A study with Dutch people found an increase in loneliness among frail people over three years ^25^ ; in another study with Mexicans living in the United States, 16% reported that they “almost never” had anyone to talk to, tell or discuss their problems ^7^ . A cross-sectional study in Korea indicates that 11% to 15% of individuals had no one to talk to or count on ^27^ . 

 The need to connect is a human characteristic and is directly associated with bonds and feelings of companionship ^28^ , so that not having children, relatives, or friends can generate feelings of loneliness. It is even possible that one of the causes of persistent negative social interactions experienced by older adults is the mismatch between the need for social support they require and the capacity of members of their social network to provide it, which would produce tensions ^1^ . Therefore, assessing feelings of loneliness and the quality of relationships established by older adults have to be a priority for health and social assistance services in view of the risks of frailty and future disabilities ^4^ . 

 According to the conceptual model of Berkman and Krishna ^6^ , the State is part of the macrosocial structure of individuals’ social networks. Therefore, it is its role to promote actions that expand social support, care, and assistance for these individuals and their families ^24^ . Neumann and Albert ^29^ highlight that Brazil should offer long-term care policies, given the growing number of older adults with disabilities and the reduced availability of family care. The authors emphasize the need for the State to recognize the value of care provided by families, including providing them with financial support. 

 A successful experience of instrumental and emotional social support is the *Maior Cuidado* Program in Belo Horizonte (state of Minas Gerais), which supports vulnerable families in providing home care to older people who need help to perform basic activities of daily living, preventing situations of risk, exclusion, social isolation, and family overload, in an inter-sectoral manner ^30^ . 

 Intervention in the individuals’ social network can act as a factor in modifying frailty, in addition to the interventions already described in the literature such as: physical activity, protein/calorie supplementation in cases of malnutrition and weight loss, and management of sarcopenia ^2^ . The implementation by the government of Community Groups, Day Centers, Homes, Community Housing, and programs such as *Maior Cuidado* are possible interventions in the individuals’ social network, which provide social support with an impact on the health and well-being of individuals and their families. 

This study has strengths and weaknesses. A strong point is its pioneering nature in analyzing various elements of the social network in a nationally representative sample. Nonetheless, its cross-sectional nature does not allow for establishing causal relationships between the social network and frailty. In addition, all social network variables included reflect the individuals’ perception, which does not always indicate reality. In addition, the instrumental social support questions included reflected an expectation of receiving help from other people in the event of a possible health need, which may not be consistent with the actual availability of help. Another limitation was the lack of information about the number of friends and neighbors to measure the social network size, which may have explained the higher average social network size among frail individuals, compared to non-frail and pre-frail individuals.

Thus, future studies could objectively measure the availability of support offered by social network members or count the members of the social network in relation to strong or weak ties regarding social support provision. It is worth noting that ELSI-Brazil is a prospective cohort study, and future analyses of the same elements of the social network from a longitudinal perspective may clarify the temporality of the associations found.

Finally, frailty is reinforced in its multidimensional perspective, so that interventions in the social network have the potential to reduce and prevent frailty. Public policymakers, authorities, and health and social assistance professionals should cover the older adults’ social network, in relation to the characteristics of social network ties, social support, and negative social interaction. Implementing the services already guaranteed by law needs to be an immediate action by the State as a way of supporting the family in care, expanding the possibilities of social support provided, and reducing feelings of loneliness.
